# Immunological Traits of Patients with Coexistent Inflammatory Bowel Disease and Periodontal Disease: A Systematic Review

**DOI:** 10.3390/ijerph18178958

**Published:** 2021-08-25

**Authors:** João Martins de Mello-Neto, Jessica Gomes Rodrigues Nunes, Santosh Kumar Tadakamadla, Carlos Marcelo da Silva Figueredo

**Affiliations:** 1 School of Medicine and Dentistry, Menzies Health Institute Queensland, Griffith University, Gold Coast, QLD 4222, Australia; j.martinsdemelloneto@griffith.edu.au (J.M.d.M.-N.); s.tadakamadla@griffith.edu.au (S.K.T.); 2 Department of Periodontology, Faculty of Odontology, Rio de Janeiro State University, Rio de Janeiro 20551-030, Brazil; jessanunes@gmail.com

**Keywords:** inflammatory bowel disease, ulcerative colitis, Crohn’s disease, gingivitis, periodontitis, systematic review

## Abstract

This systematic review assessed studies that evaluated the immunological traits of patients with both inflammatory bowel disease (IBD) and periodontal disease. An electronic search for literature was conducted on PubMed, Embase, Scopus, Cochrane and Web of Science. Studies that evaluated the immunological response in patients with IBD and periodontal disease were considered eligible for inclusion. A total of 6 cross-sectional studies of 275 patients were included. Immunological analyses were performed in gingival crevicular fluid, saliva, serum, intestinal and gingival biopsies. Four studies identified that the presence of IBD and periodontal disease was associated with higher levels of prostaglandin E2, aMMP8, IL-18 and S100A12, respectively, when compared to patients without the coexistence of both diseases. Furthermore, another study identified higher aMMP-8 levels with increasing severity of periodontitis in Crohn’s disease patients. The quality of overall evidence ranged from high to low due to the observational nature of contributing studies. The coexistence of IBD and periodontal disease seems to be associated with a more responsive inflammatory reaction compared with individuals having one or the other. More randomized controlled studies evaluating the coexistence of IBD and periodontitis are required to better explore the immunological interplay between them.

## 1. Introduction

Inflammatory bowel disease (IBD), an umbrella term for Ulcerative colitis (UC) and Crohn’s disease (CD), is a complex chronic inflammatory condition of the gastrointestinal tract [1]. Symptoms of IBD vary depending on the location and severity of inflammation and involve diarrhea, bleeding ulcers, stomach pain, cramping, bloating, weight loss and anemia, which might cause a debilitating condition with social and economic impacts [2]. Despite limited epidemiological data from developing nations, both the incidence and prevalence of IBD are increasing worldwide [3]. The pathophysiology behind IBD has been under intense research, and much of it is still unknown. Nevertheless, currently, the disease is the result of an inappropriate immune response against environmental factors, including luminal and microbial antigens, in genetically susceptible hosts [1].

Evidence has shown that IBD patients are at higher risk of periodontitis and are more frequently edentulous than controls [4,5]. In addition, recent systematic reviews with meta-analyses have also confirmed the association between IBD and periodontitis [4,6,7,8]. Periodontitis occurs as an aberrant immunological response against the constant polymicrobial biofilm challenge, leading to attachment loss and, in severe cases, tooth loss [9]. Its severe form, which can lead to tooth loss, affects around 796 million people worldwide, making it the 11th most prevalent disease in human beings [10,11].

The persistence and dysregulation of the host immune and inflammatory responses are mainly responsible for the tissue destruction in both IBD and periodontal disease [12,13], and the inflammatory response activation might be the link between them [14]. In fact, the activity of IBD influences the inflammatory response not only in the intestinal tissue but also causes systemic inflammation as demonstrated by the higher levels of TNF-α and IL-17 in the blood of patients with IBD in comparison to patients without IBD or those in disease remission [15,16,17]. Regarding periodontal disease, patients with severe periodontitis have elevated levels of inflammatory mediators (such as IL-1, IL-6, C-reactive protein (CRP) and fibrinogen) and increased neutrophil numbers in the blood when compared with healthy controls, contributing to the perpetuation of the patient’s inflammatory state [18,19,20]. These observations have generated the hypothesis that IBD and periodontal diseases have a bidirectional relationship where one disease might worsen the other [21]. Hajishengallis and Chavakis [18] have highlighted that oral pathobiont-reactive T cells (enriched in Th17 cells), which expand during periodontitis, migrate through the lymphatics to the gut, where they are activated by the ectopically colonized oral pathobionts upon their processing by antigen-presenting cells. In fact, an important *in vivo* study has found that oral inflammation, such as ligature induced periodontitis, exacerbates gut inflammation by supplying the gut with both colitogenic pathobionts and pathogenic T cells [22]. Recently, a scoping review has assessed the microbiological and immunological association between both diseases [23]. However, this is the first systematic review to solely investigate the immunological pathway. Therefore, the present systematic review was conducted to answer a clearly formulated question: Does the coexistence of IBD and periodontal disease influence the traits of the immunological response?

## 2. Materials and Methods

The reporting of this review complies with the Preferred Reporting Items for Systematic Reviews and Meta-Analyses (PRISMA) statement guidelines [24]. The study was registered in PROSPERO (REF: CRD42021254507).

### 2.1. Literature Search Strategy

Two independent reviewers (JMMN and JGRN) conducted an electronic search through December 21, 2020, using Medical Subject Headings and other free terms or keywords on PubMed to access Medline. The search strategy was adapted to other electronic databases, including Embase, Scopus, Cochrane and Web of science; search was restricted to articles published in English. Appropriate Boolean operators (OR, AND) were used to refine the searches. Search strategy used in PubMed was: (((((inflammatory bowel diseases) OR (ulcerative colitis)) OR (Crohn’s disease)) OR (Crohns disease)) OR (Colitis))) AND ((((((((((periodontal diseases) OR (periodontitis)) OR (aggressive periodontal disease)) OR (periodontitis aggressive)) OR (chronic periodontitis)) OR (chronic periodontal disease)) OR (gingival diseases)) OR (gingivitis)) OR (teeth loss)) OR (tooth loss) AND (english[Filter])

The search strategy of the other databases is presented in the Supplementary Material S1. A further manual search was conducted by reviewing the reference lists of the relevant review articles. The titles and abstracts of the resulting articles were independently screened by two reviewers (JMMN and JGRN). Those articles found to be relevant on abstract screening were reviewed in full. Disagreement between reviewers was resolved through discussion. When an agreement could not be reached, a third reviewer (CMSF) was consulted. Pre-piloted forms were independently used for data extraction by two researchers (JMMN and JGRN), which was checked for accuracy by a third reviewer (CMSF). Data on the study design, matched variables, participants (sex distribution, age, ethnicity, other systemic diseases), periodontal disease definition, type of assay, immunological analyses, sample type, collection tool, storage temperature, immunological outcomes included in the study and their effect on periodontal outcomes were extracted. When important data in the retrieved articles were missing, an attempt was made to contact the authors.

### 2.2. Focus Question

In accordance with the PE (I) CO framework [24], we used the focus question, “Does the coexistence of IBD and Periodontitis influences the traits of the immunological response?”

- Population: IBD patients, periodontal disease patients and patients with both diseases
- Exposure/Intervention: Coexistence of periodontal disease and IBD
- Comparison: Subjects with solely IBD or periodontal disease or healthy controls
- Outcomes: Immunological evaluation

### 2.3. Inclusion Criteria

Case-control, cross-sectional, longitudinal, and cohort studies in humans that evaluated immunological changes in gingival crevicular fluid, blood, saliva, feces and/or biopsies of patients with IBD and periodontitis were considered for inclusion.

### 2.4. Exclusion Criteria

Studies that did not evaluate immunological changes in patients with periodontitis and IBD and studies that evaluated only the immunological outcomes in IBD or periodontal diseases alone. Conference abstracts, opinion pieces, reviews and editorials were also excluded.

### 2.5. Assessment of Bias within Studies

As only cross-sectional studies ended up being included in this systematic review, the original Newcastle–Ottawa Scale (NOS maximum 9 stars) was modified (maximum 8 stars) to assess the quality of these studies [25]. The detailed assessment of quality for all included studies was conducted by two reviewers independently (JMMN and JGRN) based on the NOS criteria adapted for cross-sectional studies by Zhao et al. [25]. Any discrepancies were resolved by discussion. The following study aspects were evaluated: representativeness of the sample, sample size, ascertainment of exposure, non-response rate, comparability, assessment of the outcome and statistical analysis. Each cross-sectional study could be awarded a maximum of eight stars. If a study fulfilled the description followed by a star, then this symbol was assigned to that study. Therefore, a percentage score based on the overall stars awarded was calculated. Those with 80% or higher scores were classified as high methodological quality, 51–79% scores were graded as moderate quality, and the studies with scores of 50% or lower were deemed to be of low quality [26].

Data were pooled into tables, and a descriptive summary was created to determine the quantity of data and study variations (characteristics and results). The information from the included studies was tabulated according to study designs, subject characteristics, sample characteristics, cytokines investigated and the main outcomes.

## 3. Results

**Figure 1:**
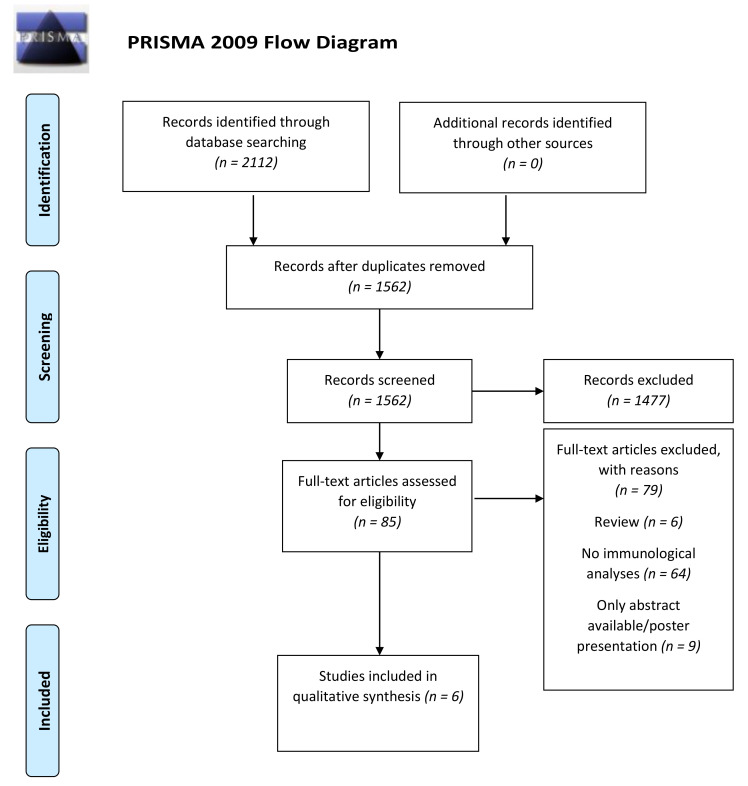
PRISMA Flowchart of the online databases searched and selection of studies for inclusion.

The electronic search of the databases identified a total of 2112 papers as follows: Medline (*n* = 347), Embase (*n* = 672), Scopus (*n* = 686), Cochrane (*n* = 17), Web of Science (*n* = 390). All the relevant articles identified through the manual search were observed in the articles retrieved through the electronic search. After the elimination of duplicates and analyses of the titles and abstracts, 85 full texts were analyzed (Figure 1). Finally, six articles were included in the qualitative analysis [27,28,29,30,31,32]. Supplementary Material Table S1 depicts the excluded studies. The Kappa test statistic was 1.0 for the studies analyzed, indicating no disagreement between the reviewers.

### 3.1. Background Characteristics of the Included Studies

In total, six cross-sectional studies were included in this systematic review. A total number of 275 individuals were included in the reviewed studies. Of those, 176 were diagnosed with IBD (166 exhibit IBD and periodontal disease and 10 IBD patients without a diagnosis of periodontal disease). In the remaining 99 patients, 89 had periodontal disease only, and 10 were healthy controls. The studies assessed immunological changes in subjects in GCF [27,28,29], serum [27,28], saliva [30], gingival biopsies [31,32] and intestinal biopsies [31,32]. Different methods of analysis (three studies used ELISA assay while Luminex assay and radioimmunoassay were used by two and one study, respectively) were used to evaluate the role of cellular immune response in the presence of both IBD and periodontitis. Analysis of the geographic distribution revealed that four studies were carried out in Brazil [28,30,31,32] and one each in the United States [27] and Germany [29]. None of the studies performed a power calculation. Four studies identified that the coexistence of IBD and periodontal disease is associated with higher levels of pro-inflammatory molecules when compared to patients with one or the other [27,28,29,30]. One study showed that the activity of IBD increases the levels of IL-4, IL-10 and IL-21in the periodontal tissue [31]. A second study showed higher levels of Interleukin (IL)-17A, IL-17F, IL-22, IL-25, IL-33, Interferon (INF)-g and IL-10 in gingival tissue, as well as a tendency for higher levels of IL-6, IL-31 and IL-21 when compared with intestinal tissue [32]. Table 1 summarizes the included studies that evaluated possible common immunological features between IBD and periodontal disease. Table 2 highlights the periodontal findings. Due to the high methodological and clinical heterogeneity between the studies, it was not possible to perform a meta-analysis.

### 3.2. Results from Individual Studies

- Immunological changes in GCF

The immunological analysis of GCF was performed in three studies [27,28,29]. One study detected Prostaglandin E2 (PGE2) levels four times higher in the group with IBD and periodontal diseases when compared to the matched periodontitis patients [27]. A second study identified higher levels of aMMP-8 in severe periodontitis patients with CD compared to UC and healthy controls also with severe periodontitis. Indeed, in CD aMMP-8, values increased significantly with increasing severity of periodontitis [29]. A third one has shown that the total amount of IL-4 was significantly lower in shallow sites from IBD patients with periodontitis compared to non-IBD patients with periodontitis [28].

- Systemic Immunological changes

The systemic immunological evaluation in the presence of both diseases was performed in two studies. One study identified higher levels of IL-8 in serum of patients with both diseases compared to periodontitis only [28]. In addition, a second study also demonstrated the chemotactic response of normal neutrophils to be significantly inhibited by serum from patients with IBD and periodontal disease. The chemotactic response of normal neutrophils was not affected by serum from healthy donors. Regarding IBD patients without periodontal disease, there were mixed results, with two of six Crohn’s disease patients and two of four ulcerative colitis patients exhibiting defects. In addition, patients with Ulcerative colitis exhibited reduced chemotactic response [27].

- Salivary changes in patients with IBD and periodontitis

Only one study with a high risk of bias evaluated immunological changes in the saliva of patients having both diseases [30]. Higher expression of S100A12 was found in patients with UC with chronic periodontitis when compared to non-IBD and CD patients also with periodontitis [30].

- Subdivision of IBD patients with periodontitis into Ulcerative colitis and Crohn’s disease

Although UC and CD are collectively known as IBD, the comparisons of immunological responses between these two distinct IBD entities (UC and CD) has been reported among all six studies. Van Dyke et al. pointed out that the chemotactic response of neutrophils in serum from patients with CD who had periodontal disease was not different from healthy subjects. However, patients with UC exhibited reduced chemotactic response [27]. Regarding IBD patients without periodontal disease, there were mixed results, with two of six CD patients and two of four UC patients exhibiting reduced chemotactic response. Figueredo et al. have also recently shown that salivary levels of S100A12 were significantly higher in patients with UC compared to CD and non-IBD patients [30]. On the other hand, Figueredo et al. highlighted increased levels of IL-23 in gingival tissue in patients with CD in comparison to patients with UC [31]. Schmidt et al. also found that patients with severe periodontitis and CD show the highest aMMP-8 concentration compared to UC and healthy controls [29]. A fifth study also found immunological differences when comparing CD and UC patients also with periodontitis. CD patients showed a strong, significant positive correlation between IL-6 in gingival crevicular fluid and IFN-y in serum, and UC patients showed a significant, positive correlation between IL-1β in GCF and IL-18 in serum [28]. However, other authors in a study without controls did not find immunological differences between UC and CD patients also with periodontitis [32].

- Immunological changes in gingival and intestinal tissues

Two studies evaluated immunological changes in intestinal and gingival tissues [31,32]. High levels of IL-17A, IL-17F IL-22, IL-25, IL-33, IL-10 and INF-y were found in gingival biopsies compared with intestinal biopsies from patients with IBD and periodontitis [32]. Figueredo et al. found that the IBD activity (evaluated by chemical and laboratory parameters) significantly increased the expression of IL-4, IL-10 and IL-21 in gingival tissue of patients with periodontitis [31]. Regarding intestinal tissue analyses, higher levels of IL-1β, IL-4, IL-6, IL-17A, IL17F, IL-21, IL-31, IL-33 and soluble CD40 ligand (sCD40L) were found in patients having IBD activity and periodontitis [31].

### 3.3. Risk of Bias (Quality Assessment)

Of the six studies included in this systematic review, one received a 3-point score out of a total of 8 points (low methodologic quality) [30]; three received 6-point scores [28,31,32]; two received 7-point scores (high quality) [27,29]; (Supplementary Material Table S2). Although none of the studies performed a sample calculation, one study showed a sample with high representativeness of periodontitis and IBD [29]. All studies described standardized, conventional immunological methods i.e., ELISA assay, Multiplex, chemotaxis and phagocytosis assay, but none reported blinding of examiners. All but one study [30] considered in this review reported adequate assessment of periodontal diseases conditions using clinical periodontal parameters (BOP, CAL, PD, GBI, CPITN); however, three studies did not report how the diagnosis of IBD was confirmed [27,28,31].

## 4. Discussion

The systemic impact of the coexistence of IBD and periodontitis has been evaluated in two studies [27,28]. Figueredo et al. showed that higher levels of IL-18 in serum of patients with both diseases when compared to patients with periodontitis only [28]. It is known that IL-18, together with IL-1β, not only contributes to the host’s defense against infections by activating phagocytes, such as monocytes, macrophages, dendritic cells and neutrophils, but also induces T-helper 17 (Th17)- and Th1-mediated adaptive immune responses [33]. However, the absence of a group with IBD and without periodontitis enables more comparisons as higher levels of IL-18 have been previously found in the plasma of patients with IBD, especially in CD [34]. Besides, Van Dyke et al. identified important alterations in neutrophils chemotactic response in the presence of both diseases [27]. It has been shown that dysfunctional neutrophil chemotaxis predisposes patients with periodontitis to neutrophil-mediated collateral host tissue damage [35]. In addition, one of the most obvious features in inflamed mucosa in IBD patients during the acute stage is the formation of crypts and abscesses, which results from the influx of neutrophils into the epithelial area and subsequently into the intestinal lumen [36]. Thus, chemotactic changes caused by the coexistence of IBD and periodontitis may impact the pathogenesis of both diseases. However, more studies are needed to confirm the systemic impacts of the coexistence of IBD and periodontitis.

Regarding the local impact of periodontitis, previous research has shown that decreased levels of IL-4 and increased levels of MMP-8, PGE2 and S100A12 in the oral cavity are associated with higher periodontal deterioration [37,38,39,40]. IL-4 is an anti-inflammatory cytokine that functions mainly by suppressing the pro-inflammatory milieu [41]. IL-4 plays a regulatory role in inhibiting Th1 responses, which are typified by cellular immune responses to intracellular pathogens [42]. Karttunen et al. [43] have previously reported that the frequency of IL-4-secreting mucosal cells is lower in IBD patients compared with controls. Thus, the significantly lower GCF levels of IL-4 in deep sites of IBD patients with periodontitis when compared with non-IBD patients with periodontitis may be associated with important immunological alterations when both diseases coexist [28]. Besides that, PGE2 regulates many physiological functions of the gut, including mucosal protection, gastrointestinal secretion and motility, inhibits fibroblast migration in intestinal wound healing and is implicated in the pathophysiology of IBD [44]. In the intestine of patients with IBD, aMMP8 are elevated and associated with neutrophil infiltration [45]. Taking together, the higher levels of aMMP-8 and PGE2 identified with the coexistence of IBD and periodontitis and lower levels of IL-4 seems to alter immunological traits in GCF negatively. In addition, one study with a higher risk of bias highlighted higher expression of S100A12 in patients with UC and chronic periodontitis. S100A12 is a well-known biomarker used to differentiate IBD from Inflammatory bowel syndrome, and its levels are higher serum and feces of IBD patients [46,47]. Moreover, S100A12 is highly abundant in neutrophils during acute inflammation and has been implicated in immune regulation and IBD pathogenesis. Therefore, the increased salivary levels of S100A12 in patients with UC and periodontitis deserves to be further explored.

The evaluation of disease activity is critical in order to monitor and adjust therapy in patients with IBD [48]. Only one study evaluated the influence of IBD activity in the presence of periodontitis [31]. The IBD activity was associated with significantly increased expression of IL-4, IL-10 and IL-21 in the gingival tissue of patients with periodontitis. Regarding intestinal tissue analyses, higher levels of IL-1β, IL-4, IL-6, IL-17A, IL17F, IL-21, IL-31, IL-33 and sCD40L were found in patients with IBD and periodontitis. These data add to evidence that IBD activity also affects the immunological response in the gingival tissue, which also merits further investigation concerning whether one disease can be affecting the progression of the other. However, a limitation of this study is the absence of a control group with one or no disease enabling further conclusions if the coexistence of both the diseases alters patient immunological traits.

The role of immunity and, in particular, the Th17 pathway in the periodontitis-IBD relationship has been recently highlighted by Kitamoto et al. [22]. The authors have shown that ligature induced periodontitis generates oral pathobiont-reactive Th17 cells that can migrate to the inflamed gut. These Th17 cells of oral origin can be activated by translocated oral pathobionts and cause the development of colitis when in the gut through IL-1β [22]. Indeed, Byrd et al. [14] have hypothesized that IBD can be driven by microbiomial and inflammatory changes originating specifically from the gingival niche through saliva, thereby worsening IBD outcomes and thus perpetuating a vicious cycle. In fact, a recent scoping review has found that periodontitis and IBD patients share similarities in their microbiological and immunological patterns [23]. However, we are the first to highlight through a systematic review the immunological changes that occur when IBD and periodontitis coexist in the same patient.

Besides, a higher inflammatory response in patients with periodontitis has been previously linked to the development of colorectal precursor lesions in observational studies [49]. The plausible mechanistic links between periodontal disease and cancer, includes chronic low-grade systemic inflammation, increased exposure to free radicals and active intermediates causing oxidative/nitrosative stress, but no mechanisms have been established yet, and more research may help gain a better understanding in this regard [50,51,52].

There are some limitations to our analysis. First, all six studies included in this systematic review are cross-sectional studies, and they cannot establish temporality. Furthermore, they do not help determine the cause–effect relationship between the diseases. Besides, our parameter here was based only on the total amount/concentration of given molecules. Second, the sample size in the included studies is small, limiting the generalizability of their findings. Third, four of the six studies come from the same research group, highlighting limited diversity of the origin of the studies. In addition, the lack of homogenous quantitative data for meta-analysis could be considered a drawback of the present systematic review. Fourth, the variability in diagnostic criteria for periodontitis and in the consideration of different potential confounders such as smoking, medications and activity of IBD could diminish the precision of this systematic review.

## 5. Conclusions

The coexistence of IBD and periodontal disease seems to be associated with a more responsive inflammatory reaction when compared with individuals having IBD or periodontal disease alone. On the basis of current data, the coexistence of both diseases might exacerbate the inflammatory response, and, therefore, more tissue destruction in both periodontal and intestinal tissue might be expected. More well-designed longitudinal studies with larger sample sizes and control groups, evaluating the immunological response as well as the impact of its treatment in the progression of both diseases, are required. Furthermore, confirmatory studies should be performed to evaluate if other inflammatory biomarkers are involved in the pathogenesis of both diseases.

## Figures and Tables

**Table 1:** Characteristics of the included studies.

Author (Year)	Matched Variables	Participants	Medicaments/Smoking Habits/Disease Activity	Periodontal and IBD Diagnose	Type of Essay	Sample	Main Immunological Outcomes
Van Dyke et al., 1986	Age and gender.	IBD patients with (*n* = 10) and without (*n* = 10) periodontal disease Patients with periodontitis (*n* = 8) Healthy controls (*n* = 8)	Steroid therapy Subjects were not included if they smoked ≤ 10 cigarettes daily. No disease activity information	Gingival erythema, edema, suppuration, BOP, pain upon probing, Ramfijord attachment levels and Periapical X-ray. IBD: combination of symptoms, including diarrhea, abdominal pain, bleeding, weight loss, perianal disease, and arthritis. Control: healthy individuals who had no bone loss or no/mild gingivitis.	Radioimmunoassay: PGE2 Phagocytosis: Van Furth method Boyden chamber assay. Neutrophils Chemotaxis assay.	GCF Serum	PGE2 sig. > in GCF of IBD patients with periodontitis compared to matched adult periodontitis. Chemotactic response sig < in UC patients with periodontal disease compared to healthy patients. The chemotactic response of normal neutrophils was significantly inhibited by all serum from patients with IBD and periodontal disease. The chemotactic response of normal neutrophils was not affected by serum from normal donors. IBD patients without the periodontal disease had mixed results. Neutrophil phagocytosis was similar between the groups.
Figueredo et al., 2011	Age, gender.	CD patients with periodontitis (*n* = 15) UC patients with periodontitis (*n* = 15) Periodontitis patients (*n* = 15)	CD: immunomodulators (*n* = 7), aminosalicylates (*n* = 4) and immunomodulators + aminosalicylates (*n* = 2). UC: immunomodulators (*n* = 1), aminosalicylates (*n* = 9) and immunomodulators + aminosalicylates (*n* = 5). CD (*n* = 3), UC (*n* = 1) and controls (*n* = 2) were smokers. UC: active disease (*n* = 3) and 12 in remission. CD: active disease (*n* = 5) remission (*n* = 10).	At least five inflamed sites with PD of ≥5 mm and CAL of ≥3 mm in different teeth and diagnosed with chronic untreated Periodontitis. CD and UC: outpatients already diagnosed with IBD attending the Gastroenterology clinics.	ELISA for IL-18. Multiplex assay for IL-1β, IL-4, IL-6, IL-10, IL-12p40, IL-12p70, TNF-α and IFN-γ.	GCF Serum	IL-4 sig. < in deep sites of patients with CD and periodontitis compared to periodontitis only. UC patient < total amount of IL-4 in the GCF in the shallow site and a > IL-6 in deep sites, when compared with periodontitis only. Serum levels of IL-18 sig. > in patients with the coexistence of IBD and periodontitis. Positive correlation between IL-6 in GCF and IFN-γ in serum of CD patients. Positive correlation between IL-1β in gingival crevicular fluid and IL-18 in serum of UC patients. No correlation was observed in periodontitis only.
Figueredo et al., 2017	Age and gender.	DC patients with periodontitis (*n* = 10) UC patients with periodontitis (*n* = 11) No control group without IBD.	Mesalazine (*n* = 7), mesalazine + azathioprine (*n* = 8), mesalazine, azathioprine + TNF-α inhibitor (*n* = 4) and mesalazine + steroid (*n* = 2). Smoking habits not reported CD (*n* = 4) and UC (*n* = 4) had active disease and CD (*n* = 6) and UC (*n* = 7) were in remission.	At least 10 teeth with PD ≥ 5 mm and CAL ≥ 4 mm in at least 4 sites, in different teeth. IBD: clinical, endoscopic, radiologic, and histological parameters.	Multiplex assay for IL-1β, IL-4, IL-6, IL-10, IL-21, IL-22, IL-23, IL-25, IL-31, IL-33, IL-17A, IL-17F, IFN-γ, sCD40L, and TNF-α.	Gingival and Intestinal tissues	Cytokine levels were similar in intestinal tissue between CD and UC patients. IL-23 in gingival tissue in CD sig. > compared to UC patients with periodontitis. IL-4, IL-10, and IL-21 levels in gingival tissue sig. > inactive IBD. Furthermore, a trend towards > IL-1β levels. IL-1β, IL-4, IL-6, IL-17A, IL17F, IL-21, IL-31, IL-33, and sCD40L were sig. > in intestinal tissue from patients with active disease. Furthermore, a trend towards increased levels of TNF-α inactive IBD.
Schmidt et al., 2018	Age and gender.	CD patients with periodontal disease (*n*= 30) UC patients with periodontal disease (*n*= 29) Controls (*n* = 59)	Aminosalicylates and azathioprine (14%), aminosalicylates and anti-TNF (5%), immunomodulators and anti-TNF (3%), corticosteroids and anti-TNF (5%). Corticosteroids and aminosalicylates and corticosteroids and immunomodulator (methotrexate) (2%). Immunomodulators (22% azathioprine/6-mercaptopurine, 2% methotrexate, 2% cyclosporine). No information available IBD (*n* = 5). CD (*n* = 14) and 20 periodontal disease (*n* = 20) were smokers. Disease activity information available (*n* = 33), UC (*n* = 3) active and *n* = 30 on remission	Periodontal condition was classified into healthy/mild, moderate or severe Periodontitis. No assessment of bleeding on probing (BOP). Papilla bleeding index (PBI). IBD: outpatients recruited within a private gastroenterological clinic.	ELISA assay for aMMP-8	GCF	aMMP-8 in IBD sig. > compared to control. No sig. difference between CD and UC. aMMP-8 in CD sig. > with increasing severity of periodontitis. Periodontitis severity had no influence on aMMP-8 in UC and controls. aMMP-8 > in patients with severe periodontitis and CD compared to UC and HC also with severe periodontitis. aMMP-8 in CD patients with no or mild periodontitis was < than in the case of UC.
Manegat et al., 2016	Age and gender.	DC patients with periodontitis (*n* = 18) UC patients with periodontitis (*n* = 10) No control group without IBD.	CD: immunomodulators (*n* = 13), 5-aminosalicylate derivatives (*n* = 11), corticosteroids (*n* = 1), immunomodulatory derivatives + 5-aminosalicylate (*n* = 10) no medication (*n* = 4). UC: 5-aminosalicylate derivatives (*n* = 8), immunomodulators (*n* = 5), corticosteroids (*n* = 1) and immunomodulatory derivatives + 5-aminosalicylate acid derivatives (*n* = 4) no medication (*n* = 1). CD group had two smokers and one ex-smoker and UC had three ex-smokers. CD (*n* = 1) and UC (*n* = 2) with disease active.	At least 8 teeth with PD ≥ 5 mm and CAL ≥ 4 mm in at least 4 sites, in different teeth. IBD: clinical, endoscopic, radiologic, and histological parameters.	LUMINEX for IFN-γ, IL-1β, IL-4, IL-6, IL-10, IL-21, IL-22, IL-23, IL-25, IL-31, IL-33, IL-17A, IL-17F, sCD40L, and TNF-α	Gingival and intestinal tissues	No differences in cytokine levels between CD and UC patients in the gingival tissue. IL-17A, IL-17F, IL-22, IL-25, IL-33, INF-g, and IL-10 sig. > in gingival tissue. IL-6, IL-31 and IL-21 in gingival tissue showed a tendency to > levels compared to intestinal tissue.
Figueredo et al., 2021	Age, gender.	CD patients with periodontitis (*n* = 9) UC patients with periodontitis (*n* = 9). Periodontitis patients (*n* = 5).	Not reported	Not reported	ELISA for S100A12	Saliva	S100A12 sig. > in patients with UC patients with periodontitis compared to CD and non-IBD patients also with periodontitis.

aMMP-8:Active Matrix Metalloproteinase-8; BOP: Bleeding on probing; BI: bleeding index; CAL: Clinical attachment loss; CD: Crohn’s Disease; CPITN: Community Index of Periodontal Treatment Needs; CRP: C-reactive protein; GCF: gingival crevicular fluid; GBI: Gingival bleeding index; HC: Healthy controls; IBD: Inflammatory bowel disease; IFN: interferon; IL: interleukin; MCP−1: Monocyte Chemoattractant Protein-1; VPI: Visible plaque index; PBI: papilla bleeding index; PI: Plaque index; PGE2: Prostaglandin E2; PD: Probing depth; sCD40L: soluble CD40 ligand; SD: Standard deviation; Sig: significant; TNF: Tumor necrosis factor; UC: Ulcerative colitis.

**Table 2:** Periodontal characteristics of the included studies.

Author (Year)	Periodontal Findings
Van Dyke et al., 1986	Comparison of IBD periodontitis and periodontitis groups revealed no significant differences in CAL. Periodontal health of the IBD patient is one of moderate to severe periodontitis with extreme inflammation compared with periodontitis only. Redness Bleeding, CAL and PPD were significant > in IBD with periodontitis and adult periodontitis compared to IBD with no periodontitis and controls.
Figueredo et al., 2011	CAL, BOP, PI and GCF volume did not differ between the groups.
Figueredo et al., 2017	PI, BOP, PD, CAL did not differ between the groups.
Schmidt et al., 2018	Significant higher CAL was found in IBD compared to HC, but no significant difference was found for PPD. The PBI was significantly higher in IBD than in HC. IBD group significantly had more participants with severe periodontitis compared to HC. Periodontal parameters did not significantly differ between CD and UC.
Figueredo et al., 2021	Not reported
Menegat et al., 2016	CD patients presented higher % PD significant > 7 mm compared to UC patients also with periodontitis.

BOP: Bleeding on probing; BI: Bleeding index; CAL: Clinical attachment loss; CD: Chron’s Disease; HC: Healthy controls; IBD: Inflammatory bowel disease; PBI: Papilla bleeding index; PI: Plaque index; PPD: Probing pocket depth.

## Supplementary Materials

The following are available online at https://www.mdpi.com/article/10.3390/ijerph18178958/s1, S1: Database Search Strategies, Table S1: Reasons for excluding after assessing eligibility criteria., Table S2: Risk assessment of included studies.
