# Supplementary material for: Immunological Traits of Patients with Coexistent Inflammatory Bowel Disease and Periodontal Disease: A Systematic Review
Source: Int J Environ Res Public Health. 2021 Aug 25;18(17):8958. doi: 10.3390/ijerph18178958 (PMC8430503; doi:10.3390/ijerph18178958)
Supplement: Supplementary file 1 [file ijerph-18-08958-s001.zip › Supplementary material 1_ Search strategy.pdf]

Supplementary material S1 - Database Search Strategies

PUBMED: (((((inflammatory bowel diseases) OR (ulcerative colitis)) OR (Crohn´s disease)) OR (Crohns disease)) OR (Colitis))) AND ((((((((((periodontal diseases) OR (periodontitis)) OR (aggressive periodontal disease)) OR (periodontitis aggressive)) OR (chronic periodontitis)) OR (chronic periodontal disease)) OR (gingival diseases)) OR (gingivitis)) OR (teeth loss)) OR (tooth loss) AND (english[Filter])

EMBASE: 'inflammatory bowel disease' OR (ulcerative AND colitis) OR (crohn´s AND disease) OR 'crohn disease' OR colitis) AND ('periodontal disease' OR periodontitis OR 'aggressive periodontitis' OR 'chronic periodontitis' OR (chronic AND periodontal AND disease) OR 'gingiva disease' OR gingivitis OR (teeth AND loss) OR (tooth AND loss)) AND ([english]/lim).

COCHRANE: Inflammatory bowel disease OR Ulcerative Colitis OR Colitis OR Chron´s Disease OR Crohn Disease in All Text AND Periodontal disease OR periodontitis OR Aggressive periodontitis OR Chronic periodontitis OR Gingival disease OR gingivitis OR Teeth loss OR tooth loss.

SCOPUS: inflammatory bowel diseases OR ulcerative colitis OR crohn's disease OR crohn disease OR colitis AND Periodontal diseases OR periodontitis OR Aggressive periodontitis OR Chronic periodontitis OR Gingival disease OR Gingivitis OR Teeth loss OR Tooth loss.

WEB OF SCIENCE: Inflammatory bowel diseases OR ulcerative colitis OR crohn's disease OR crohn disease OR colitis AND Periodontal diseases OR periodontitis OR Aggressive periodontitis OR Chronic periodontitis OR Gingival disease OR Gingivitis OR Teeth loss OR Tooth loss.
