# Supplementary material for: Immunological Traits of Patients with Coexistent Inflammatory Bowel Disease and Periodontal Disease: A Systematic Review
Source: Int J Environ Res Public Health. 2021 Aug 25;18(17):8958. doi: 10.3390/ijerph18178958 (PMC8430503; doi:10.3390/ijerph18178958)
Supplement: Supplementary file 1 [file ijerph-18-08958-s001.zip › Supplementary material_Table 1. Reasons for excluding after assessing eligibility criteria. .pdf]

Supplementary Table S1 – Reasons for excluding after assessing eligibility criteria.

ONLY ABSTRACT/POSTER PRESENTATION

	AUTHOR	YEAR	Title
1	Ahmed, O.	2013	The prevalence of dental disease in inflammatory bowel disease - American Journal of Gastroenterology
2	Barreiro-de Acosta, M.	2016	Oral manifestations in inflammatory bowel disease patients under anti-tumour necrosis factor treatment
3	Kelsen, J.	2013	Pediatric crohn's disease intrinsic associations with the subgingival microbiota revealed by a prospective longitudinal cohort study
4	Kim, J. S.	2019	Increased risk of ulcerative colitis in patients with periodontitis: a nationwide populationbased study
5	Koutsochristou, V.	2014	Profiles of dental caries and periodontal disease in individuals with or without inflammatory bowel disease (IBD)
6	Piatek, D	2018	Selected cytokines in periodontitis in patients with Crohn's disease-preliminary results from polibd study
7	Piatek D	2018	The impact of Crohn's disease on periodontal status-preliminary results from polibd study
8	Docktor, M	2010	The oral microbiome in children with inflammatory bowel disease
9	Vandyke, T. E.	1984	Immunological and microbiologic characterization of periodontal-disease in crohns-disease patients

REVIEW

1	Keskin, M.	2015	Two Cheers for Crohn's Disease and Periodontitis: Beta-Defensin-2 as an Actionable Target to Intervene on Two Clinically Distinct Diseases
2	Mantegazza, C.	2016	Oral manifestations of gastrointestinal diseases in children. Part 1: General introduction
3	Mantegazza, C.	2016	Oral manifestations of gastrointestinal diseases in children. Part 3: Ulcerative colitis and gastro-oesophageal reflux disease

4	Niculescu, Z.	2010	Oral manifestations of gastrointestinal diseases: An interdisciplinary approach
5	Taylor, V. E	1975	Oral manifestations of Crohn's disease without demonstrable gastrointestinal lesions
6	Topbas, A.	2018	Early Detection of Crohn's Disease on the Basis of Oral Manifestations in a Case with Marfan Syndrome

NO IMMUNOLOGICAL ANYLISIS/PATIENTS WITH ONLY ONE DISASE

1	Brito F.	2008	Prevalence of periodontitis and DMFT index in patients with Crohn's disease and ulcerative colitis
2	Brito F	2013	Subgingival microflora in inflammatory bowel disease patients with untreated periodontitis
3	Chi YC.	2013	Increased risk of periodontitis among patients with Crohn’s disease: a population-based matched-cohort study
4	Docktor, M. J.	2012	Alterations in diversity of the oral microbiome in pediatric inflammatory bowel disease
5	Flemmig, T. F.	1991	Prevalence and severity of periodontal disease in patients with inflammatory bowel disease
6	Fumery, O.	2017	Patients with inflammatory bowel disease have an increase risk of periodontitis correlated with disease activity
7	Gieorgijewska, A.	2011	Periodontal status evaluation in patients with diagnosed IBD: Crohn's disease and ulcerative colitis
8	Harty, S.	2005	A prospective study of the oral manifestations of Crohn's disease
9	Jones, J. V.	1986	Phenotypic characteristics of motile, nonspore-forming, gram-negative anaerobes from periodontal lesions of patients with inflammatory bowel-disease
10	Kelsen, J.	2012	The subgingival oral microbiome in pediatric patients with crohn's disease

11	Kelsen, J.	2015	Alterations of the Subgingival Microbiota in Pediatric Crohn's Disease Studied Longitudinally in Discovery and Validation Cohorts
12	Kelsen, J.	2013	Longitudinal characterization of the subgingival microbiota in pediatric crohn's disease
13	Klaniecka, B.	2016	Oral Cavity Condition and Selected Salivary Parameters in Children and Adolescents Suffering from Inflammatory Bowel Diseases
14	Koutsochristou, V.	2015	Dental caries and periodontal disease in children and adolescents with inflammatory bowel disease: A case-control study
15	Kumar, K. M.	2018	Association of oral manifestations in ulcerative colitis: A pilot study
16	Laws, A. J.	1979	Interdental colitis: the entity and its management
17	Lazzerini, M.	2015	Orofacial granulomatosis in children: Think about Crohn's disease
18	Lee, A. M.	2013	Intestinal failure and home parenteral nutrition: Implications for oral health and dental care
19	Lin, C. Y.	2018	Increased risk of ulcerative colitis in patients with periodontal disease: A nationwide population-based cohort study
20	Lovelina, F. D.	2016	Oral health status of ulcerative colitis patients: A comparative study
21	Malathi, K.	2017	Gingival enlargement unveiling Crohn's disease - A report of a rarefied case
22	Mergulhao, P.	2005	Gingival hyperplasia as a first manifestation of Crohn's disease
23	Meurman, J. H.	1994	Gingival and dental status, salivary acidogenic bacteria, and yeast counts of patients with active or inactive Crohn's disease
24	Misra, S.	1996	Orofacial lesions in Crohn's disease

25	Nagpal, S.	2012	Periodontal disease and anemias associated with Crohn's disease. A case report
26	Nemetz, A	2001	Allelic variation at the interleukin 1 β gene is associated with decreased bone mass in patients with inflammatory bowel diseases
27	Nishizawa, T.	2019	Clinical features of very early-onset inflammatory bowel disease in Japan, a single centre pilot study
28	Ojha, J.	2007	Gingival involvement in Crohn disease
29	Oostlander, A. E.	2012	T cell-mediated increased osteoclast formation from peripheral blood as a mechanism for crohn's disease-associated bone loss
30	Plauth, M.	1991	Oral manifestations of Crohn's disease. An analysis of 79 cases
31	Pujol Muncunill, G.	2019	Oral and periodontal manifestations in Paediatric Inflammatory Bowel Disease
32	Rio Rouco, P.	2015	Oral and periodontal manifestations in Paediatric Inflammatory Bowel Disease
33	Schiller, K. F.	1971	Crohn's disease of the mouth and lips
34	Schuler, D.	1971	[Ulcerative hyperplastic gingivitis in one member of a pair of monozygotic twins]
35	Schulz, S.	2014	Tumor necrosis factor-α and oral inflammation in patients with Crohn disease
36	Sigusch, B. W.	2004	Periodontitis as manifestation of Crohn's disease in primary dentition: A case report
37	Simpson, H. E	1974	Oral manifestations of Crohn's disease
38	Sternthal, M. B.	2008	Adverse events associated with the use of cyclosporine in patients with inflammatory bowel disease
39	Ursomanno, B. L.	2019	Treatment of Crohn's disease and Ulcerative colitis with proton pump inhibitors: effect on bone loss at dental implants

40	Vavricka, S. R.	2013	Periodontitis and Gingivitis in Inflammatory Bowel Disease: A Case-Control Study
41	Vinesh, E.	2016	A Clinicopathologic Study of Oral Changes in Gastroesophageal Reflux Disease, Gastritis, and Ulcerative Colitis
42	Vonaesch, P.	2018	Stunted childhood growth is associated with decompartmentalization of the gastrointestinal tract and overgrowth of oropharyngeal taxa
43	Ward, C. S.	1985	Crohn's disease limited to the mouth and anus
44	Wiesenfeld, D	1985	Oro-facial granulomatosis--a clinical and pathological analysis
45	Wray, D.	1998	Gastrointestinal and granulomatous diseases
46	Yerke, L	2019	Relationship between proton pump inhibitors and periodontal disease: Treatment considerations for IBD patients
47	Yin, W.	2017	Inverse Association Between Poor Oral Health and Inflammatory Bowel Diseases
48	Yu, H. C.	2018	Inflammatory bowel disease as a risk factor for periodontitis under Taiwanese National Health Insurance Research database
49	Zhang, L.	2020	Increased risks of dental caries and periodontal disease in Chinese patients with inflammatory bowel disease
50	Cotti, E.	2018	Healing of Apical Periodontitis in Patients with Inflammatory Bowel Diseases and under Anti-tumor Necrosis Factor Alpha Therapy
51	de Vries, S. A. G.	2018	Salivary Function and Oral Health Problems in Crohn's Disease Patients
52	Francis, D. L.	2018	Oral health status of ulcerative colitis patients: A comparative study
53	Gemmell, M. R.	2017	Defining genome diversity of campylobacter concisus between oral, faecal and colonic biopsy isolates

54	Grošelj, D.	2008	Prediction of clinical response to anti-TNF treatment by oral parameters in Crohn's disease
55	Holmes, A.	1985	Gingival swelling as the presenting feature of Crohn's disease in children
56	Hujoel, P. P.	2016	Dental Morbidities, Smoking, Oral Hygiene, and Inflammatory Bowel Diseases
57	Johannsen, A.	2015	Consumption of Dental Treatment in Patients with Inflammatory Bowel Disease, a Register Study
58	Kang, E. A.	2019	Increased risk of Ulcerative colitis in patients with periodontitis: a nationwide population-based study
59	Kang, E. A.	2020	Periodontitis combined with smoking increases risk of the ulcerative colitis: A national cohort study
60	Kato, I.	2020	History of inflammatory bowel disease and self-reported oral health: Women's health initiative observational study
61	Xu, A A	2020	Oral Health and the Altered Colonic Mucosa-Associated Gut Microbiota
62	Said, H S	2014	Dysbiosis of Salivary Microbiota in Inflammatory Bowel Disease and Its association with Oral Immunological Biomarkers
63	Stein, J. M.	2010	Clinical periodontal and microbiologic parameters in patients with crohn's disease with consideration of the CARD15 genotype
64	Simpson, H. E.	1976	Oral manifestations of Crohn's disease
