# Supplementary material for: Immunological Traits of Patients with Coexistent Inflammatory Bowel Disease and Periodontal Disease: A Systematic Review
Source: Int J Environ Res Public Health. 2021 Aug 25;18(17):8958. doi: 10.3390/ijerph18178958 (PMC8430503; doi:10.3390/ijerph18178958)
Supplement: Supplementary file 1 [file ijerph-18-08958-s001.zip › Supplementary material_Table 2. Risk assessement .pdf]

Supplementary Table S2. Quality Assessment of cross-sectional studies included (Newcastle-Ottawa scale modified by D. Zhao et al. (2018)).

	SELECTION (Max. 4*)				COMPARABILITY (max. 2*)	OUTCOME (Max. 2*)		Total starts (max. 8*)
	Representativeness of the sample	Sample size	Ascertainment of exposure	Non-response rate		Assessment of outcome	Statistical analysis	
VAN DYKE, 1986	*		*	*	**	*	*	7
Figueredo et al., 2011	*		*	*	*	*	*	6
Menegat et al., 2016	*		*	*	*	*	*	6
Schmidt et al., 2018	*	*	*	*	*	*	*	7
Figueredo et al., 2021			*	*			*	3
Figueredo et al., 2017	*		*	*	*	*	*	6
